# Mental health in individuals with spinal cord injury: The role of socioeconomic conditions and social relationships

**DOI:** 10.1371/journal.pone.0206069

**Published:** 2019-02-20

**Authors:** Carmen Zürcher, Hannah Tough, Christine Fekete, for the SwiSCI Study Group

**Affiliations:** 1 Bern University of Applied Sciences, Department of Health Professions, Bern, Switzerland; 2 Swiss Paraplegic Research, Nottwil, Switzerland; 3 University of Lucerne, Department of Health Sciences and Health Policy, Lucerne, Switzerland; Northwestern Medicine, UNITED STATES

## Abstract

**Objectives:**

To evaluate socioeconomic inequalities in social relationships, and to assess whether socioeconomic conditions and social relationships are independently related to mental health problems in individuals with a physical disability due to spinal cord injury (SCI).

**Methods:**

We analyzed cross-sectional data from 511 individuals with SCI aged over 16 years who participated in the community survey of the Swiss SCI Cohort Study (SwiSCI). Indicators for socioeconomic conditions included years of formal education, household income, and financial strain. Social relationships were operationalized by three structural (partner status; social contact frequency; number of supportive relationships) and four functional aspects (satisfaction with: overall social support; family relationships; contacts to friends; partner relationship). General mental health was assessed by the Mental Health Inventory (MHI-5) of SF-36 and depressive symptoms were measured by the Hospital Anxiety and Depression Scale (depression subscale, HADS-D). Established cut-offs for general mental health problems (MHI-5 ≤56) and depressive symptomatology (HADS-D ≥8) were used to dichotomize outcomes. Associations were assessed using logistic regressions.

**Results:**

Lower household income was predominantly associated with poor structural social relationships, whereas financial strain was robustly linked to poor functional social relationships. Financial strain was associated with general mental health problems and depressive symptomatology, even after controlling for social relationships. Education and household income were not linked to mental health. Poor structural and functional social relationships were related to general mental health problems and depressive symptomatology. Notably, trends remained stable after accounting for socioeconomic conditions.

**Conclusion:**

This study provides evidence for socioeconomic inequalities in social relationships as well as for independent associations of financial strain and poor social relationships with mental health problems in individuals with SCI. Further research may develop strategies to improve mental health in SCI by strengthening social relationships. Such interventions may be especially beneficial for individuals with low income and financial strain.

## Introduction

The World Health Organization estimates that about 10% of the world’s population is affected by mental health disorders [1] and the prevalence is even higher in individuals with disabilities [2,3]. Spinal cord injury (SCI) is a condition that often causes major physical disability, as the damage to the spinal cord leads to a total or partial loss of sensation and movement below the lesion level [4]. An increased risk of mental health disorders has also been observed in individuals with SCI [5–9]. Especially, depression and anxiety disorders are common following SCI with prevalence rates of about 22% [5] and 27% [10], respectively. In light of these figures, it is vitally important to better understand the factors that are related to mental health in the SCI population in order to inform tailored interventions and health policies.

Socioeconomic conditions [11–14] and social relationships [11,14–16] have been identified as potential determinants of mental health. Evidence from general population samples indicates that individuals in unfavorable socioeconomic conditions have a higher risk for mental health disorders than better situated individuals [17–21]. Socioeconomic inequalities in mental health have also been shown in the SCI population [22–25]. Likewise, there is evidence from general [26,27] and SCI populations [28,29] that individuals with poor social relationships are more likely to experience mental health problems than those with adequate structural and functional social relationships. *Structural* measures thereby describe quantitative aspects of social relationships (e.g., network size, frequency of social contacts), whereas *functional* measures represent qualitative aspects (e.g., social support, satisfaction with relationships) [30,31]. This distinction is essential since earlier research has suggested that functional aspects of social relationships are more important for mental health than structural aspects [26,28,29].

Although the link of socioeconomic conditions and social relationships with mental health is well established, less is known on socioeconomic inequalities in social relationships. Literature suggests that individuals with unfavorable socioeconomic conditions are more likely to have poor social relationships compared to those who are better situated [32–34]. It thus seems important to study socioeconomic inequalities in social relationships as such interrelations might confound the suggested associations of socioeconomic conditions and social relationships with mental health.

**Fig 1:**
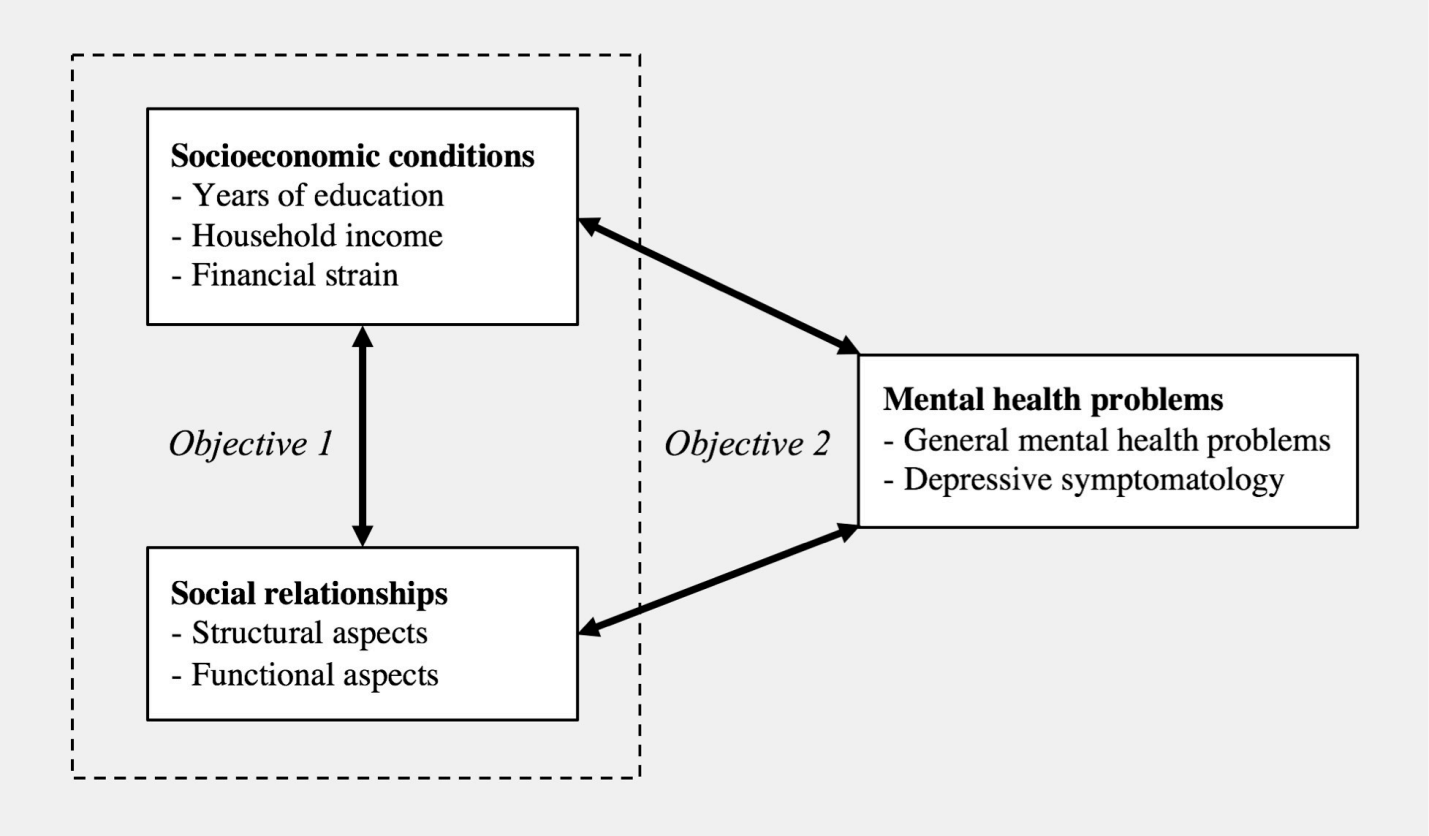
Analytical framework.

To date, neither the association between socioeconomic conditions and social relationships, nor their independent association with mental health have been investigated in the SCI population. Therefore, the study objectives are (1) to evaluate socioeconomic inequalities in structural and functional aspects of social relationships, and (2) to explore whether socioeconomic conditions and social relationships are independently related to mental health problems in Swiss residents with SCI (Fig 1).

## Methods

### Study design

We analyzed data from the first cross-sectional community survey of the Swiss Spinal Cord Injury Cohort Study (SwiSCI) [35]. The survey consisted of three modules (a starter, a basic, and one out of three thematically specific modules) which were subsequently distributed in intervals of about three months [35,36]. The data were collected between late 2011 and early 2013 by means of paper-and-pencil forms, online questionnaires or telephone interviews [37]. The absolute response rate of the starter module was 61.1%, and cumulative response rates for the basic and the specific modules were 49.7% and 42.7%, respectively [37]. Further details on the study design, recruitment outcomes and non-response are described elsewhere [35–37]. The study was approved by the Ethics Committee of the Canton Lucerne, Switzerland, and all participants signed a written consent form. Given the self-report nature of this survey, participants capacity to consent could not be verified. The SwiSCI Steering Committee accepted the scientific proposal for the present study.

### Sample

The survey included Swiss residents aged at least 16 years with traumatic or non-traumatic SCI. Individuals with SCI due to congenital conditions, neurodegenerative disorders, Guillain-Barré syndrome and those in palliative care settings were excluded [35]. The sampling frame consisted of individuals drawn from address lists of the Swiss Paraplegic Association, the specialized home care institution ParaHelp, and three Swiss SCI rehabilitation centers [35,37]. Of a total of 3144 eligible individuals, 1331 completed all three modules [36,37]. For this study, we used data from 511 individuals who participated in the thematically specific ‘Psychological Personal Factors and Health Behavior’ module.

### Measures

Socioeconomic conditions: Years of formal education, net-equivalent household income, and financial strain were selected as indicators for socioeconomic conditions. In accordance with the International Standard Classification of Education’s definition [38], education was measured combining years of schooling and vocational training. The monthly net-household income in Swiss Francs (CHF) was assessed on a seven-point scale. We then weighted the mean of the respective response by household size and structure to obtain the net-equivalent household income according to the Organization for Economic Co-operation and Development’s modified scale [39]. Financial strain was measured by asking the respondents about problematic financial situations restricting their life during the past four weeks. Response options were ‘not applicable’, ‘had no impact’, ‘slightly complicated my life’, ‘massively complicated my life’. For analysis, the categories ‘not applicable’ and ‘had no impact’ were combined and classified as ‘no financial strain’. We introduced education per year and household income per CHF 1000 as continuous variables into all models, and used the information on financial strain as a categorical indicator.

Social relationships were operationalized by three structural (partner yes/no; social contact frequency; number of supportive relationships), and four functional aspects (satisfaction with: overall social support; family relationships; contacts to friends; partner relationship). The psychometrically validated six-item short form of the Social Support Questionnaire (SSQ6) was used to assess the number of supportive relationships and the satisfaction with overall social support [40]. The first part of each question assessed the number of supportive relationships on a scale ranging from 0–9, while the second part captured the support satisfaction on a six-point scale [40]. Mean sum scores were constructed for number of supportive relationships. For the satisfaction items, mean sum scores were calculated if at least three of the six items were completed. Based on distribution-based quintiles of the average number of supportive relationships, we built a dichotomous variable for analyses on socioeconomic inequalities in social relationships (lowest quintile being coded as ‘few supportive relationships’ vs. higher quintiles being coded as ‘several supportive relationships’). For analyses on social relationships and mental health, the number of supportive relationships was entered into the model as a continuous variable. Satisfaction with overall social support was dichotomized into ‘less than satisfied’ vs. ‘satisfied’.

The validated Utrecht Scale for Evaluation of Rehabilitation-Participation (USER-P) [41–43] was used to assess social contact frequency and satisfaction with different social relationships. Participants were asked how many times they had visited or had been visited by family members or friends, and had been in contact with others using a telephone or computer within the past four weeks on a six-point scale ranging from ‘never’ to ‘19 times or more’. The average frequency of weekly contacts was dichotomized into ‘infrequent’ for the lowest distribution-based quintile and ‘frequent’ for higher quintiles for analyses on socioeconomic inequalities in social relationships. For analyses on social relationships and mental health, average frequency of weekly contacts was entered into the model as a continuous variable. Further, three USER-P items assessed the satisfaction with family relationships, contacts to friends, and the partner relationship on a five-point scale ranging from ‘very dissatisfied’ to ‘very satisfied’ with the option ‘not applicable’ for partner relationship satisfaction [41]. Equal to the item on overall social support satisfaction, each item was dichotomized into ‘less than satisfied’ vs. ‘satisfied’.

General mental health problems were assessed with the five-item Mental Health Inventory (MHI-5), a subscale of the 36-item Short Form Health Survey (SF-36) [44]. The MHI-5 showed good reliability and validity as screening instrument for general mental health problems in functionally impaired individuals [45,46]. The participants rated the frequency of emotional states during the past four weeks on a six-point scale from ‘all of the time’ to ‘none of the time’. The raw sum scores were transformed to a 0–100 scale [47]. We dichotomized the scale based on recommendations for mental health monitoring in Europe (≤56 general mental health problems; >56 no general mental health problems) [48–50].

Depressive symptomatology was assessed with the depression subscale of the Hospital Anxiety and Depression Scale (HADS-D) [51], its use in the SCI population being supported by several studies [52,53]. The HADS-D comprises seven four-point scaled items on pleasure experiences in the past week [54]. The sum score ranging from 0–21 was dichotomized (≥8 for depressive symptomatology) according to the literature [51].

The strategy of dichotomization of general mental health and depressive symptoms was chosen as the aim of this study was to investigate whether unfavorable socioeconomic conditions and poor social relationships independently increase the odds for relevant mental health *problems* and less so whether there was a dose-response relationship between socioeconomic conditions, social relationships and mental health outcomes.

Control variables: Based on literature, we included self-report measures on sex [9,55,56], age [23,55,56], level and degree of lesion (complete/incomplete paraplegia, complete/incomplete tetraplegia) [8,57], etiology (traumatic/non-traumatic) [58,59], time since injury [57,60,61], chronic pain [29,62,63], and paid employment [9,64,65] as potential confounders into multivariate analyses. Chronic pain was assessed with an item on the frequency and severity of pain problems, rated on a four-point scale ranging from 0 ‘no or insignificant problem’ to 4 ‘frequent or chronic problem’. Persons who indicated having some pain were classified as having pain. Paid employment was measured with an item on the current employment status, asking participants whether they were currently engaged in paid work or not.

### Statistical analyses

After frequency analyses of main study variables, we investigated unadjusted and adjusted associations between socioeconomic conditions, social relationships, and mental health using logistic regressions. To assess the association between socioeconomic conditions and social relationships, we ran unadjusted (model 1) and confounder-adjusted (model 2) regressions of social relationship aspects on socioeconomic conditions. In model 2, indicators of socioeconomic conditions were not mutually adjusted (e.g., models for education were not adjusted for financial strain and income). To investigate whether socioeconomic conditions and social relationships were related to mental health, mental health was regressed on socioeconomic conditions and social relationships. Again, we conducted unadjusted analyses (model 1) and analyses adjusted for the potential confounders sex, age, level and degree of lesion, etiology, time since injury, chronic pain, paid employment (model 2). Beside adjustment for potential confounders, regressions on socioeconomic conditions were additionally adjusted for all social relationship variables (model 3a), and regressions on social relationships were additionally adjusted for all indicators of socioeconomic conditions (model 3b) to assess their *independent* associations with mental health. In model 3a, socioeconomic conditions were not mutually adjusted, and in model 3b, social relationship variables were not entered simultaneously into the model for mutual adjustment. To evaluate the association of partner relationship satisfaction with mental health, only those who identified having a partner were included in the respective analyses.

Frequency analyses were performed on full case data, whereas techniques to account for unit and item non-response were adopted for regression analyses [66]. We adjusted for unit non-response by introducing inverse probability weights (IPWs) that were derived from available information on basic sociodemographic and lesion characteristics of the sampled population [36]. To deal with item non-response, we applied multiple imputation using chained equations to impute predictors and potential confounders [67]. To get sample-appropriate multiply imputed data, we chose the number of imputations according to the fraction of missing information (FMI) [67]. For the first and the second set of analyses, 15 and 35 datasets were imputed, respectively. Full case data was used for the outcome variables.

For each outcome, we reported odds ratios (OR), 95% confidence intervals (95% CI), and *p* values of unrestricted FMI tests as proportions of missing values were unequally distributed across variables [68]. *P* values of ≤0.05 were considered statistically significant. In case of the categorical variable on financial strain, global tests were additionally performed to assess the overall significance of the variable (results only reported in the text).

Bonferroni corrections were applied as sensitivity analyses to address the issue of multiple testing. The following threshold of Bonferroni-adjusted *p* values have been considered as significant on an alpha 5% level: *p*≤0.002 for analyses on socioeconomic inequalities in social relationships (28 adjusted model 2 tests); *p*≤0.006 for analysis on socioeconomic inequalities in mental health (8 adjusted model 3a tests); *p*≤0.004 for analysis on social relationships and mental health (14 adjusted model 3b tests).

We further computed variation inflation factors (VIFs) to scan for multi-collinear predictors and treated VIFs below 10 as unproblematic [69].

To test the robustness of the results, we performed sensitivity analyses comparing four distinct scenarios regarding the handling of missing values, each with an unweighted and a weighted model. The four scenarios included: (1) multiply imputed data for predictors and control variables, and full cases for outcomes; (2) full cases for all included variables; (3) multiply imputed data for predictors and control variables, and replacement of missing values in outcomes by ‘best case’ values; (4) multiply imputed data for predictors and control variables, and replacement of missing variables in outcomes by ‘worst case’ values. This paper reports the results of the weighted scenario 1. As a further sensitivity analysis to test the robustness of the dichotomization of mental health variables, we used tobit models to assess the associations of social relationships and socioeconomic conditions with continuous mental health outcomes. The sensitivity analyses comparing the distinct scenarios and the tobit models are reported in the Supporting information File (S1 File).

All analyses were conducted using Stata version 14.2 for Mac (College Station, StataCorp LP) [70].

## Results

Sample characteristics are shown in Table 1. Of the 511 participants, nearly three quarters were male and the mean age was around 53 years. About two thirds of the study population had paraplegia, and slightly more than half indicated having an incomplete lesion. Most SCI cases were due to traumatic events, and the mean time since injury was around 17 years. Almost three in four participants reported experiencing chronic pain. The participants had a mean formal education of around 14 years and a monthly net-equivalent household income of roughly CHF 4200. One third of the study population reported financial strain that slightly or massively complicated their life in the prior four weeks, and nearly 43% stated being in paid employment. One in three participants had no partner. The average social contact frequency was about six to seven times per week and participants in the lowest quintile were categorized as having infrequent social contacts (less than three per week). The number of supportive relationships averaged nearly three. Again, participants in the lowest quintile were categorized as having few supportive relationships (one and a half or less). About 7% were less than satisfied with their overall social support, while around one out of six was not satisfied with family relationships or contacts to friends. Of those participants who reported having a partner, almost one fifth was less than satisfied with their partner relationship. Based on MHI-5 and HADS-D scores, around one fifth of the population was classified as showing general mental health problems or depressive symptomatology, respectively.

**Table 1:** Basic characteristics of the SwiSCI study population (N = 511).

Characteristic [measure]	[m]	n (%)	Mean (SD); median (IQR)
Sociodemographic and lesion characteristics			
Sex	0		
Female		140 (27.4)	
Male		371 (72.6)	
Age in years	0		52.9 (14.8); 53.0 (21.0)
In paid employment	2	217 (42.6)	
Level and degree of lesion	3		
Complete paraplegia		166 (32.7)	
Incomplete paraplegia		184 (36.2)	
Complete tetraplegia		56 (11.0)	
Incomplete tetraplegia		102 (20.1)	
Etiology	2		
Traumatic		400 (78.6)	
Non-traumatic		109 (21.4)	
Time since injury in years	5		17.4 (13.1); 14.0 (19.4)
Chronic pain	26	350 (72.2)	
Socioeconomic conditions			
Years of formal education	0		13.8 (3.3); 13.0 (4.0)
Net-equivalent household income (CHF)	48		4195.9 (1915.4); 3750.0 (2750.0)
Financial strain	22		
No strain		327 (66.9)	
Slight strain		118 (24.1)	
Massive strain		44 (9.0)	
Structural aspects of social relationships			
Not having a partner	19	163 (33.1)	
Social contact frequency, per week [USER-P]	33		6.3 (3.6); 6.5 (5.1)
Infrequent social contacts (lowest quintile)		99 (20.7)	
Number of supportive relationships, range 0–9 [SSQ-6]	46		2.9 (1.8); 2.7 (2.2)
Few supportive relationships (lowest quintile)		110 (23.7)	
Functional aspects of social relationships			
Satisfaction with support and relationships			
Less than satisfied with overall social support [SSQ-6]	24	34 (7.0)	
Less than satisfied with family relationships [USER-P]	24	79 (16.2)	
Less than satisfied with contacts to friends [USER-P]	21	82 (16.7)	
Less than satisfied with partner relationship [USER-P]	31^a^	53 (17.8)	
Mental health			
General mental health, range 0–100 [MHI-5]	55		72.5 (17.8); 76.0 (28.0)
General mental health problems (score ≤56)		102 (22.4)	
Depression, range 0–21 [HADS-D]	13		4.6 (3.9); 4.0 (5.0)
Depressive symptomatology (score ≥8)		106 (21.3)	

*Abbreviations*: *HADS-D*: Hospital Anxiety and Depression Scale, subscale depression; *IQR*: Inter-quartile range; *m*: Number of missing values; *MHI-5*: 5-item Mental Health Inventory of SF-36; *SD*: Standard deviation; *SSQ-6*: Social Support Questionnaire, 6-item short form; *USER-P*: Utrecht Scale for Evaluation of Rehabilitation-Participation

^a^ Of participants having a partner

### Socioeconomic conditions and social relationships

Results on socioeconomic inequalities in social relationships are displayed in Table 2. Education was inconsistently related to social relationships, whereas household income was associated with four out of seven aspects of social relationships. More specifically, participants with higher household income were less prone having infrequent social contacts (OR 0.83, 95% CI 0.71–0.99, *p*≤0.05), few supportive relationships (OR 0.86, 95% CI 0.75–0.99, *p*≤0.05) and lower satisfaction with their partner relationship (OR 0.81, 95% CI 0.67–0.98, *p*≤0.05) than those with higher household income. Lower household income was tentatively linked to dissatisfaction with overall social support and family relationships (*p*>0.05). However, we observed that individuals with a higher household income were more likely to have no partner (OR 1.13, 95% CI 1.00–1.26, *p*≤0.05).

**Table 2:** Associations of socioeconomic conditions with structural and functional aspects of social relationships, odds ratios (OR) and 95% confidence intervals (95% CI) of logistic regressions.

	Structural aspects of social relationships			Functional aspects of social relationships			
	Not having a partner	Infrequent social contacts^a^	Few supportive relationships^b^	Less than satisfied with overall social support	Less than satisfied with family relationships	Less than satisfied with contacts to friends	Less than satisfied with partner relationship
Number of observations	492	478	465	487	487	490	298
Effect size	*OR (95% CI)*	*OR (95% CI)*	*OR (95% CI)*	*OR (95% CI)*	*OR (95% CI)*	*OR (95% CI)*	*OR (95% CI)*
**Years of formal education (per year)**							
Model 1	0.96 (0.91–1.02)	0.94 (0.87–1.00)	0.97 (0.90–1.05)	0.91 (0.81–1.01)	1.01 (0.94–1.09)	1.01 (0.93–1.11)	1.01 (0.92–1.10)
Model 2	0.97 (0.92–1.04)	0.95 (0.88–1.02)	1.00 (0.93–1.08)	0.92 (0.81–1.03)	1.04 (0.95–1.13)	1.05 (0.95–1.16)	1.00 (0.90–1.11)
**Net-equivalent household income (per CHF 1000)**							
Model 1	1.08 (0.96–1.20)	0.84 (0.72–0.97)*	0.86 (0.76–0.98)*	0.91 (0.74–1.12)	0.94 (0.82–1.07)	0.94 (0.82–1.08)	0.83 (0.69–0.99)*
Model 2	1.13 (1.00–1.26)*	0.83 (0.71–0.99)*	0.86 (0.75–0.99)*	0.98 (0.77–1.25)	0.95 (0.82–1.11)	1.00 (0.85–1.16)	0.81 (0.67–0.98)*
**Financial strain**							
No strain (Reference)	1.00	1.00	1.00	1.00	1.00	1.00	1.00
Model 1							
Slight Strain	1.36 (0.86–2.17)	1.40 (0.83–2.38)	1.07 (0.63–1.82)	1.79 (0.76–4.23)	1.76 (0.98–3.19)	1.55 (0.88–2.75)	1.97 (0.96–4.04)
Massive Strain	1.52 (0.76–3.01)	1.61 (0.76–3.42)	1.86 (0.88–3.93)	5.32 (2.08–13.57)***	5.70 (2.76–11.76)***	3.59 (1.72–7.48)**	5.41 (2.01–14.55)**
Model 2							
Slight Strain	1.48 (0.91–2.41)	1.23 (0.72–2.09)	1.08 (0.61–1.92)	1.42 (0.59–3.41)	1.69 (0.87–3.28)	1.50 (0.80–2.83)	2.37 (1.12–5.08)*
Massive Strain	1.38 (0.69–2.74)	1.64 (0.72–3.70)	2.25 (0.98–5.12)	5.18 (1.90–14.09)**	5.84 (2.59–13.17)***	3.15 (1.42–6.99)**	8.46 (2.87–24.94)***

* *p* ≤ 0.05

** *p* ≤ 0.01

*** *p* ≤ 0.001. *P* values from unrestricted fraction missing information tests.

*Note*: Predictors imputed by multiple imputation. Outcome variables full case, except variable on overall social support satisfaction. All analyses weighted by inverse probability weights.

^a^ Infrequent social contacts: lowest quintile of social contact frequency

^b^ Few supportive relationships: lowest quintile of number of supportive relationships.

*Model 1*: Unadjusted.

*Model 2*: Adjusted for sex, age, level and degree of lesion, etiology, time since injury, chronic pain, and paid employment. Not mutually adjusted for other socioeconomic variables.

The severity of experiencing financial strain was gradually and consistently linked to all functional aspects of social relationships. As compared to persons not reporting financial strain, the odds of poor functional relationships were significantly increased in persons reporting any financial strain (all results from global tests *p*≤0.05). While we observed tentative trends of increased odds of poor functional relationships in individuals experiencing slight financial strain, participants reporting massive financial strain were clearly more likely to be dissatisfied with their overall social support (OR 5.18, 95% CI 1.90–14.09, *p*≤0.01), family relationships (OR 5.84, 95% CI 2.59–13.17, *p*≤0.001), contacts to friends (OR 3.15, 95% CI 1.42–6.99, *p*≤0.01), and partner relationship (OR 8.46, 95% CI 2.87–24.94, *p*≤0.001) in comparison to those not reporting financial strain. Furthermore, we observed a non-significant mostly gradual trend towards infrequent social contacts, few supportive relationships and not having a partner in individuals who reported slight or massive financial strain (all *p*>0.05; Table 2).

### Socioeconomic conditions, social relationships and mental health problems

Education and household income were not significantly associated with mental health when accounting for potential confounders and social relationships. However, participants who experienced financial strain were more likely to report general mental health problems and depressive symptomatology, even after controlling for social relationships (Table 3, Model 3a; all results from global tests *p*≤0.05). The odds of depressive symptomatology was increased in persons reporting slight financial strain (OR 2.13, 95% CI 1.18–3.85, *p*≤0.05) and was highest in persons with massive financial strain (OR 2.47, 95% CI 1.13–5.41, *p*≤0.05), as compared to persons without financial strain. Similarly, in comparison to persons without financial strain, persons with massive financial strain had increased odds of general mental health problems (OR 3.11, 95% CI 1.34–7.23, *p*≤0.01).

**Table 3:** Associations of socioeconomic conditions and social relationships with mental health problems, odds ratios (OR) and 95% confidence intervals (95% CI) of logistic regressions.

		General mental health problems (MHI-5 score ≤56)	Depressive symptomatology (HADS-D score ≥8)
Number of observations		456	498
Effect sizes		*OR (95% CI)*	*OR (95% CI)*
**Socioeconomic conditions**			
**Years of formal education** (per year)	Model 1	1.01 (0.93–1.09)	0.95 (0.88–1.03)
	Model 2	1.03 (0.95–1.12)	0.99 (0.91–1.07)
	Model 3a	1.03 (0.95–1.12)	0.98 (0.92–1.05)
**Net-equivalent household income** (per CHF 1000)	Model 1	0.95 (0.84–1.08)	0.87 (0.76–0.98)*
	Model 2	0.99 (0.87–1.14)	0.90 (0.78–1.04)
	Model 3a	1.00 (0.87–1.15)	0.90 (0.77–1.05)
**Financial strain**			
No financial strain	Reference	1.00	1.00
Slight strain	Model 1	1.80 (1.04–3.11)*	2.48 (1.47–4.15)**
Massive strain		5.57 (2.75–11.32)***	4.46 (2.16–9.20)***
Slight strain	Model 2	1.69 (0.94–3.02)	2.37 (1.36–4.12)**
Massive strain		4.94 (2.41–10.12)***	4.01 (1.85–8.70)***
Slight strain	Model 3a	1.50 (0.80–2.80)	2.13 (1.18–3.85)*
Massive strain		3.11 (1.34–7.23)**	2.47 (1.13–5.41)*
**Structural aspects of social relationships**			
**Partner status**			
Having a partner	Reference	1.00	1.00
Not having a partner	Model 1	1.67 (1.04–2.68)*	1.83 (1.15–2.91)*
	Model 2	1.67 (1.01–2.76)*	2.08 (1.27–3.40)**
	Model 3b	1.62 (0.96–2.72)	2.04 (1.22–3.42)**
**Social contacts frequency**	Model 1	0.90 (0.84–0.96)**	0.93 (0.86–1.01)
	Model 2	0.88 (0.82–0.95)**	0.94 (0.87–1.02)
	Model 3b	0.87 (0.81–0.94)***	0.95 (0.88–1.03)
**Number of supportive relationships**	Model 1	0.84 (0.73–0.97)*	0.72 (0.62–0.85)***
	Model 2	0.83 (0.71–0.96)*	0.74 (0.62–0.87)***
	Model 3b	0.83 (0.70–0.98)*	0.75 (0.63–0.89)**
**Functional aspects of social relationships**			
**Satisfaction with overall social support**			
Satisfied	Reference	1.00	1.00
Less than satisfied	Model 1	3.08 (1.36–6.98)*	3.06 (1.40–6.69)*
	Model 2	2.96 (1.27–6.89)*	2.79 (1.22–6.42)*
	Model 3b	2.46 (0.95–6.37)	2.25 (0.95–5.32)
**Satisfaction with family relationships**			
Satisfied	Reference	1.00	1.00
Less than satisfied	Model 1	4.21 (2.43–7.30)***	3.44 (2.00–5.92)***
	Model 2	4.03 (2.26–7.18)***	3.25 (1.82–5.80)***
	Model 3b	3.21 (1.72–6.00)***	2.62 (1.44–5.00)***
**Satisfaction with contacts to friends**			
Satisfied	Reference	1.00	1.00
Less than satisfied	Model 1	7.48 (4.33–12.93)***	4.61 (2.70–7.89)***
	Model 2	6.60 (3.77–11.55)***	3.92 (2.24–6.88)***
	Model 3b	5.89 (3.27–10.62)***	3.45 (1.93–6.18)***
**Satisfaction with partner relationship**			
Subgroup: Number of observations		294	320
Satisfied	Reference	1.00	1.00
Less than satisfied	Model 1	4.69 (2.34–9.41)***	2.24 (1.09–4.59)*
	Model 2	6.29 (2.79–14.18)***	2.68 (1.20–5.98)*
	Model 3b	5.53 (2.44–12.51)***	2.20 (0.98–4.96)

* *p*≤0.05

** *p*≤0.01

*** *p*≤0.001. *P* values from unrestricted fraction missing information tests.

*Note*: Predictors imputed by multiple imputation, outcome variables full case only. All analyses weighted by inverse probability weights.

*Abbreviations*: *HADS-D*: Hospital Anxiety and Depression Scale, depression subscale; *MHI-5*: 5-item Mental Health Inventory of SF-36.

*Model 1*: Unadjusted.

*Model 2*: Adjusted for sex, age, level and degree of lesion, etiology, time since injury, chronic pain, paid employment. Not mutually adjusted for other socioeconomic or social relationship variables.

*Model 3a*: Model 2 additionally adjusted for functional and structural aspects of social relationships.

*Model 3b*: Model 2 additionally adjusted for socioeconomic conditions.

With respect to structural social relationships, four out of six associations with mental health problems were significant (Table 3, Model 3b). Not having a partner was related to depressive symptomatology (OR 2.04, 95% CI 1.22–3.42, *p*≤0.01), higher social contact frequency was related to lower odds of general mental health problems (OR 0.87, 95% CI 0.81–0.94, *p*≤0.001) and more supportive relationships were associated with decreased odds of general mental health problems (OR 0.83, 95% CI 0.70–0.98, *p*≤0.05*)* and depressive symptomatology (OR 0.75, 95% CI 0.63–0.89, *p*≤0.01). These associations remained stable after controlling for potential confounders and socioeconomic conditions. We also observed elevated odds for general mental health problems in participants without a partner and slightly decreased odds for depressive symptomatology in individuals with higher social contact frequency. However, these associations were just above the conventional level of statistical significance (all *p*>0.05).

Five out of eight associations between functional aspects of social relationships and mental health problems remained significant after controlling for potential confounders and socioeconomic conditions (Table 3, Model 3b). More specifically, low satisfaction with family relationships (OR 3.21, 95% CI 1.72–6.00, *p*≤0.001), contacts to friends (OR 5.89, 95% CI 3.27–10.62, *p*≤0.001), and the partner relationship (OR 5.53, 95% CI 2.44–12.51, *p*≤0.001) were related to general mental health problems. Moreover, two aspects of poor functional social relationships were still associated with depressive symptomatology after adjustment for socioeconomic conditions (low satisfaction with family relationships: OR 2.62, 95% CI 1.44–5.00, *p*≤0.001; contacts to friends: OR 3.45, 95% CI 1.93–6.18, *p*≤0.001). In addition, we observed tentative trends of increased odds for general mental health problems and depressive symptomatology in individuals with low overall social support satisfaction, and higher odds for depressive symptomatology in participants with low partner relationship satisfaction (all *p*>0.05, after controlling for socioeconomic conditions).

### Variation inflation factors and sensitivity analyses

The VIFs for all predictors and potential confounders did not indicate problematic multicollinearity (VIFs ranging from 1.08–1.57). The S1 File displays the results of the sensitivity analyses on different weighting and imputation scenarios. The trends of the scenarios were mostly consistent with the main analyses, and the statistical significance of the results varied only in few cases (indicated as bold results in the S1 File). Table C in the S1 File provides the results of the sensitivity analyses using the outcomes of mental health as continuous variables. These analyses clearly support the robustness of findings using dichotomous outcomes, indicating that less financial strain and better structural as well as functional aspects of social relationships are associated with better general mental health and less depressive symptoms (all *p*≤0.05).

Sensitivity analyses accounting for potential issues of multiple testing using Bonferrioni-adjusted significance levels indicate some vulnearbility to multiple testing. Results on socioeconomic inequalities in social relationships (Table 2, model 2) and socioeconomic inequalities in mental health (Table 3, model 3a) might be susceptible to type I error, as their *p* values were mostly above the Bonferroni-adjusted value of 0.002 and 0.006, respectively. Results on structural social relationships and mental health were predominantely above the adjusted *p* values (0.004), while results on functional relationships and mental health seemed robust against type I error (Table 3, model 3b).

## Discussion

This study is among the first to provide evidence for socioeconomic inequalities in social relationships, as well as for the independent associations of financial strain and social relationships with mental health problems in Swiss residents with a condition causing major physical disability, namely SCI. Lower household income was predominantly associated with poor structural social relationships, while financial strain was associated with poor functional social relationships. Experiencing financial strain as well as poor structural and functional social relationships was related to general mental health problems and depressive symptomatology, even after mutual adjustment for socioeconomic conditions and social relationships.

### Socioeconomic inequalities in social relationships

Previous studies largely support our findings regarding socioeconomic inequalities in social relationships [32–34], but there are some ambiguities. Earlier research suggested that an increase in education is related to better social relationships [32,33], while our results are inconclusive. In contrast to other populations, the duration of education in individuals with SCI may be affected by time-consuming occupational retraining. However, it was not specified in our questionnaire whether retraining following SCI counts as formal education or not. This discrepancy in the operationalization of education may partly explain the inconsistency of results. Future studies among individuals with acquired disability should therefore consider distinguishing between education before and after the onset of disability to retest the associations with more precise and reliable data. Alternatively, education may not have contributed to pronounced socioeconomic inequalities in our sample since the level of education was generally high.

The finding that participants with lower household income were more likely to report poor structural social relationships (i.e., infrequent social contacts and few supportive relationships) is in line with results from middle-aged and elderly general populations [32,33]. Low income may preclude individuals from establishing and maintaining social relationships by restricting social participation after SCI [71]. For example, individuals with low income may have less resources to pursue leisure activities, to join clubs, to afford specialized sports equipment, to organize transportation to events, or to invite others over. One could also argue that individuals with the lowest income are most likely not in paid employment and thus have less social networking opportunities. It is however unlikely that this explanation applies to the present context, as the strength of the association scarcely changed after controlling for employment. Against expectation, participants with a lower household income were more likely to have a partner. This finding is conflicting with other research [32] as well as with our results regarding financial strain, and therefore needs further investigation.

As financial strain has rarely been used as indicator of socioeconomic conditions, it proves difficult to directly compare our findings with the literature. One recent general population study indicated a negative association between financial strain and relationship satisfaction in couples [72]. The authors suggested that financial strain may deteriorate dyadic coping interactions through an increase in negative behaviors leading to distress in the partner relationships, and that financial strain might change the perception of how the partner fulfills his or her role in an adverse situation [72]. Another study found that financial strain mediated the association between income and relationship quality [73]. Given that financial strain seems to relate to social relationships, we recommend including financial strain as indicator of socioeconomic circumstances in future studies on inequalities in social relationships. Also, further investigations are needed to better understand the mechanisms of how financial strain might affect social relationships in individuals with a physical disability.

### Socioeconomic inequalities, social relationships and mental health problems

The finding that financial strain was consistently related to mental health, while education and household income were not, does not correspond to previous research on individuals with SCI which indicated that individuals with lower education [9,22–24,61,74,75] and lower income [22,23,25,76] were more likely to experience mental health problems. Importantly, we were not able to replicate the finding of an earlier study on the SwiSCI study population concerning educational inequalities in mental health [22] in our sub-sample. Therefore, further investigations or adaptations in the measurements are warranted. It remains unclear whether educational years were inadequately measured or were an inadequate indicator of socioeconomic conditions in the SCI population, or whether educational years played de facto no role in our sample. Concerning income inequalities, other studies also failed to observe an explicit income gradient in mental health when income was introduced as a categorical variable [23,25]. More specifically, participants with the lowest income notably had the highest risk of reduced mental health, whereas the relationships were inconsistent for higher income groups [23,25]. This may indicate that income is not linearly related to mental health, and thus did not appear relevant when introduced as continuous variable in our study. Our results further support the notion that household income and financial strain describe different socioeconomic constructs as they were differentially linked to social relationships and mental health problems. The perception of one’s financial situation might be relative to cultural and social standards [77] rather than depending on absolute income alone. Insufficient resources to satisfy personal needs may trigger feelings of relative social disadvantage that release stress reactions [19,78,79]. If chronic, such distress evidently leads to an excessive activation or dysregulation of neural and neuroendocrine responses affecting health [80,81]. One could therefore argue that financial strain is more detrimental for mental health than a low income due to its association with stress experiences.

Overall, our study provides evidence that poor social relationships were associated with mental health problems in individuals with SCI, irrespective of socioeconomic stressors. Research in the general population suggests that social relationships buffer the adverse effects of financial strain [82] and deprivation [83] on mental health. Theory furthermore posits that social relationships may affect mental health by providing social guidance and enhancing the sense of self-worth and self-efficacy, as well as supporting affective and neuroendocrine regulation [15,31,84–87]. This assumption has been supported by previous studies which have indicated positive associations of social relationships with mental health in individuals with SCI [56,74,88–96].

Our study supports the notion that structural as well as functional aspects of social relationships are associated with mental health problems. Although some associations did not remain significant after adjusting for socioeconomic conditions, no striking differences between structural or functional relationships were observed. These results were even more consistent when using the outcomes of mental health as continuous variables (cf. Table C in the S1 File). Our findings contrast the results of a recent literature review on social relationships and mental health in individuals with disabilities, showing that the functional aspects were more consistently related to mental health than structural aspects [28]. Moreover, the reviewed studies showed that structural aspects of social relationships were not related to general mental health measures but to depression [28]. Since studies on the SCI population which assess any functional aspect of social relationships other than social support are missing [28], our study addressed aspects of social relationships which were previously understudied in individuals with disabilities.

### Limitations and strengths

A major limitation of this study is its observational and cross-sectional nature, which does not allow any statements about causality. We could thus not assess if poor social relationships account for reduced mental health, or if mental health problems lead to poorer social relationships. While the comprehensive set of variables to operationalize socioeconomic conditions and social relationships can be seen as strength of the study, the resulting large number of performed statistical tests may lead to multiple testing issues, evidenced by the Bonferroni-adjustments showing vulnerability to type I error in some results. Given that the data is based on self-report, we cannot exclude social desirability bias [97]. Moreover, the IPWs did not account for a potential unit non-response bias due to socioeconomic conditions, social relationships, or mental health since the necessary data were not available for the non-responders. Therefore, the generalizability of results to the target population might be limited. Lastly, the dichotomization of the outcome variables may be criticized as loss of information. However, the aim of this study was to draw conclusions on factors associated with the occurrence of mental health problems. We thus used cut-offs for general mental health problems and depressive symptomatology that were based on internationally established recommendations.

The considerably large sample from a community survey which included at least 25% of the Swiss SCI population into its core modules is a major strength of this study [36]. The minimal unit non-response bias that was found based on basic sociodemographic and lesion characteristics was accounted for by introducing the available IPWs [36,37]. We also dealt with item non-response by means of multiple imputation. Furthermore, mental health as well as most of the social relationship aspects were assessed using items from validated instruments, and we distinguished between structural and functional aspects of social relationships to allow for a comprehensive assessment of social relationships. Finally, the sensitivity analyses comparing the distinct scenarios and the tobit models supported the robustness of our main results.

### Implications

This study emphasizes the role of financial strain and social relationships in mental health of individuals with SCI, and therefore suggests potential avenues for future intervention research that seeks to develop strategies to prevent mental health problems in persons with physical disabilities. Attenuating financial distress is likely beneficial, even in a wealthy country like Switzerland. Providing early financial advice might prevent or mitigate financial strain after SCI by accounting for the increased economic burden (e.g., uncovered health care expenditures, assistive devices). Health professionals may direct their attention to individuals in unfavorable socioeconomic conditions to prevent or appropriately treat mental health problems [98]. Cognitive behavioral therapy, for example, has been shown to enhance perceived social support and mental health after myocardial infarction [99]. Additionally, programs promoting affordable sports activities for those with low financial resources may enhance structural social relationships through improved social participation [100], and interventions to support the use of communication technologies might facilitate social integration [101]. Workshops customized to enrich relationships also seem promising in enhancing functional social relationships and therefore mental health in individuals with disabilities [102].

## Conclusion

This study provides evidence for socioeconomic inequalities in social relationships as well as for independent associations of financial strain and poor social relationships with mental health problems in individuals with SCI in Switzerland. Programs to strengthen social relationships and mental health may be especially beneficial for individuals with low income and financial strain. Yet, further efforts are needed to develop and evaluate intervention strategies to tackle potential social determinants of mental health in the SCI population.

## Supporting information

S1 File Sensitivity analyses. (PDF)
