# Supplementary material for: Mental health in individuals with spinal cord injury: The role of socioeconomic conditions and social relationships
Source: PLoS One. 2019 Feb 20;14(2):e0206069. doi: 10.1371/journal.pone.0206069 (PMC6382129; doi:10.1371/journal.pone.0206069)
Supplement: S1 File — (PDF) [file pone.0206069.s001.pdf]

Supporting information

S1 File. Sensitivity analyses.

Table A. Sensitivity analyses of associations of socioeconomic conditions with structural and functional aspects of social relationships, odds ratios (OR) and 95% confidence intervals (95% CI) of logistic regressions comparing four data scenarios, each with an unweighted (u) and a weighted (w) model.

		**Structural aspects of social relationships**			**Functional aspects of social relationships**			
		**Not having a partner**	**Infrequent social contacts**	**Few supportive relationships**	**Less than satisfied with overall social support**	**Less than satisfied with family relationships**	**Less than satisfied with contacts to friends**	**Less than satisfied with partner relationship**
		OR (95% CI)	*OR (95% CI)*	*OR (95% CI)*	*OR (95% CI)*	*OR (95% CI)*	*OR (95% CI)*	*OR (95% CI)*
**Years of formal education (per year)**								
Scenario 1	u	0.98 (0.92-1.04)	0.95 (0.88-1.02)	1.00 (0.94-1.07)	0.94 (0.83-1.06)	1.04 (0.96-1.12)	1.06 (0.98-1.15)	0.99 (0.89-1.09)
	w	0.97 (0.92-1.03)	0.95 (0.88-1.02)	1.00 (0.92-1.08)	0.92 (0.81-1.03)	1.04 (0.95-1.13)	1.05 (0.95-1.16)	1.00 (0.90-1.11)
Scenario 2	u	0.97 (0.92-1.04)	0.93 (0.86-1.01)	1.00 (0.93-1.07)	0.95 (0.84-1.08)	1.04 (0.96-1.13)	1.05 (0.97-1.14)	1.00 (0.90-1.11)
	w	0.97 (0.91-1.03)	0.94 (0.87-1.01)	1.00 (0.92-1.08)	0.92 (0.81-1.05)	1.05 (0.96-1.15)	1.04 (0.94-1.15)	1.01 (0.91-1.13)
Scenario 3	u	0.98 (0.92-1.04)	0.95 (0.88-1.02)	1.00 (0.94-1.07)	1.04 (0.96-1.12)	1.07 (0.99-1.15)	1.00 (0.92-1.10)	0.98 (0.92-1.04)
	w	0.97 (0.92-1.04)	0.95 (0.88-1.02)	1.00 (0.93-1.08)	1.04 (0.96-1.14)	1.05 (0.95-1.16)	1.02 (0.93-1.12)	0.97 (0.92-1.04)
Scenario 4	u	0.97 (0.92-1.03)	0.95 (0.89-1.01)	1.00 (0.94-1.06)	0.97 (0.89-1.06)	1.01 (0.95-1.09)	1.03 (0.96-1.11)	0.97 (0.92-1.03)
	w	0.97 (0.92-1.03)	0.95 (0.89-1.02)	0.99 (0.93-1.06)	0.95 (0.85-1.05)	1.01 (0.94-1.09)	1.02 (0.94-1.11)	0.98 (0.92-1.03)
**Net-equivalent household income (in CHF 1000)**								
Scenario 1	u	1.15 (1.03-1.28)*	0.86 (0.74-0.99)*	**0.88 (0.77-1.00)**	0.98 (0.79-1.22)	0.95 (0.82-1.10)	1.00 (0.87-1.16)	**0.82 (0.66-1.01)**
	w	1.13 (1.00-1.26)*	0.83 (0.71-0.99)*	0.86 (0.75-0.99)*	0.98 (0.77-1.25)	0.95 (0.82-1.11)	1.00 (0.85-1.16)	0.81 (0.67-0.98)*
Scenario 2	u	1.18 (1.05-1.32)**	0.83 (0.71-0.96)*	0.87 (0.75-1.00)*	0.99 (0.79-1.24)	0.97 (0.83-1.12)	1.00 (0.86-1.16)	**0.84 (0.67-1.05)**
	w	1.15 (1.02-1.30)*	0.82 (0.69-0.99)*	0.87 (0.75-0.99)*	0.99 (0.76-1.28)	0.98 (0.84-1.14)	0.99 (0.84-1.16)	**0.84 (0.68-1.03)**
Scenario 3	u	1.14 (1.02-1.27)*	0.86 (0.75-0.99)*	**0.89 (0.78-1.02)**	0.96 (0.83-1.11)	1.01 (0.87-1.17)	**0.81 (0.68-0.96)***	1.14 (1.02-1.27)*
	w	**1.12 (0.99-1.26)**	**0.86 (0.73-1.00)**	**0.90 (0.79-1.02)**	0.97 (0.83-1.12)	1.00 (0.86-1.17)	**0.81 (0.70-0.94)***	1.12 (0.99-1.26)**
Scenario 4	u	1.14 (1.03-1.27)*	0.88 (0.77-0.99)*	0.87 (0.77-0.98)*	0.87 (0.73-1.04)	0.94 (0.83-1.07)	1.00 (0.87-1.14)	**1.04 (0.94-1.16)**
	w	**1.12 (1.00-1.26)**	**0.87 (0.76-1.00)**	**0.85 (0.76-0.96)**	0.85 (0.70-1.03)	0.94 (0.81-1.08)	0.98 (0.85-1.12)	**1.03 (0.93-1.14)**
**Financial strain**								
No strain (Reference)		1.00	1.00	1.00	1.00	1.00	1.00	1.00
Scenario 1								
Slight strain	u	**1.66 (1.02-2.70)***	1.24 (0.72-2.14)	1.07 (0.61-1.88)	1.56 (0.62-3.88)	**2.11 (1.13-3.94)***	**1.87 (1.01-3.45)***	2.27 (1.03-5.01)*
Massive strain	u	1.44 (0.70-2.96)	1.44 (0.65-3.19)	2.07 (0.91-4.69)	6.13 (2.10-17.90)**	6.25 (2.80-13.92)***	3.29 (1.49-7.31)**	6.40 (2.15-19.05)**
Slight strain	w	1.48 (0.91-2.41)	1.23 (0.72-2.09)	1.08 (0.61-1.92)	1.42 (0.59-3.41)	1.69 (0.87-3.28)	1.50 (0.80-2.83)	2.37 (1.12-5.08)*
Massive strain	w	1.38 (0.69-2.74)	1.64 (0.72-3.70)	2.25 (0.98-5.12)	5.18 (1.90-14.09)**	5.84 (2.59-13.17)***	3.15 (1.42-6.99)**	8.46 (2.87-24.94)***
Scenario 2								
Slight strain	u	1.57 (0.95-2.91)	1.23 (0.68-2.20)	1.04 (0.58-1.89)	1.23 (0.46-3.30)	**2.35 (1.20-4.60***	**2.09 (1.10-3.96)***	2.53 (1.10-5.84)*
Massive strain	u	1.36 (0.64-2.90)	1.23 (0.53-2.89)	2.11 (0.92-4.83)	6.57 (2.17-19.93)**	7.99 (3.38-18.84)***	3.36 (1.45-7.78)**	7.70 (2.54-23.32)***
Slight strain	w	1.71 (1.00-2.91)	1.04 (0.57-1.88)	0.91 (0.48-1.72)	1.11 (0.40-3.06)	1.84 (0.88-3.86)	1.72 (0.97-3.41)	2.40 (1.06-5.45)*
Massive strain	w	1.63 (0.76-3.53)	1.14 (0.48-2.72)	1.96 (0.80-4.76)	5.90 (1.84-18.92)**	7.25 (2.97-17.71)***	3.16 (1.30-7.71)*	9.00 (3.01-26.95)***
Scenario 3								
Slight strain	u	**1.75 (1.08-2.85)***	1.29 (0.75-2.22)	1.13 (0.64-1.97)	1.58 (0.64-3.91)	**2.10 (1.13-3.91)***	**1.88 (1.02-3.46)***	**1.80 (0.87-3.73**
Massive strain	u	1.50 (0.73-3.07)	1.48 (0.68-3.22)	1.98 (0.91-4.32)	4.89 (1.74-13.73)**	6.17 (2.79-13.64)***	3.31 (1.51-7.25)**	3.43 (1.30-9.07)*
Slight strain	w	**1.86 (1.12-3.09)***	1.13 (0.65-1.97)	0.98 (0.54-1.79)	1.43 (0.56-3.69)	1.77 (0.89-3.52)	1.58 (0.82-3.04)	**1.73 (0.84-3.56)**
Massive strain	w	1.74 (0.85-3.64)	1.43 (0.64-3.18)	1.71 (0.75-4.03)	4.02 (1.30-12.40)*	6.06 (2.63-13.94)***	3.32 (1.43-7.70)**	3.55 (1.32-9.50)*
Scenario 4								
Slight strain	u	1.38 (0.86-2.21)	1.14 (0.68-1.94)	0.93 (0.56-1.53)	1.47 (0.71-3.06)	**2.06 (1.17-3.65)***	1.60 (0.89-2.85)	1.58 (1.00-2.49)*
Massive strain	u	1.23 (0.64-2.56)	1.35 (0.62-2.94)	1.74 (0.84-3.60)	6.01 (2.20-14.42)***	4.97 (2.31-10.55)***	2.73 (1.24-5.97)*	2.25 (1.58-6.70)**
Slight strain	w	1.48 (0.90-2.43)	1.02 (0.59-1.76)	0.88 (0.52-1.49)	1.50 (0.70-3.21)	1.74 (0.94-3.20)	1.33 (0.72-2.47)	**1.56 (0.97-2.50)**
Massive strain	w	1.53 (0.75-3.12)	1.36 (0.60-3.10)	1.79 (0.84-3.79)	6.76 (2.77-16.47)***	4.77 (2.13-10.69)***	2.71 (1.20-6.11)*	3.66 (1.71-7.80)**

* $p \leq 0.05$, ** $p \leq 0.01$, *** $p \leq 0.001$. *P* values from unrestricted fraction missing information tests for scenario 1, 3, and 4. *P* values from Wald χ^2 tests for scenario 2.
Scenario 1: Multiply imputed data for predictors and control variables, outcome variables full case; *Scenario 2*: Full cases for all included variables; *Scenario 3*: Multiply imputed data for predictors and control variables, replacement of missing values in outcomes by 'best case' values; *Scenario 4*: Multiply imputed data for predictors and control variables, replacement of missing values in outcomes by 'worst case' values.
Notes: All analyses adjusted for control variables (sex, age, level and degree of lesion, etiology, time since injury, chronic pain, paid employment). Not mutually adjusted for other socioeconomic variables. Weighted analyses conducted with inverse probability weights. **Bold** results indicate deviations from the weighted scenario 1 (main results displayed in Table 2).

Table B. Sensitivity analyses of associations of socioeconomic conditions and social relationships with mental health problems, odds ratios (OR) and 95% confidence intervals (95% CI) of logistic regressions comparing four data scenarios, each with an unweighted (u) and a weighted (w) model.

			**General mental health problems** (MHI-5 score ≤56)	**Depressive symptomatology** (HADS-D score ≥8)
			OR (95% CI)	*OR (95% CI)*
**Years of formal education (per year)**[a]	Scenario 1	u	1.04 (0.96-1.12)	0.98 (0.91-1.06)
		w	1.03 (0.95-1.12)	0.98 (0.91-1.05)
	Scenario 2	u	1.05 (0.96-1.14)	1.00 (0.92-1.10)
		w	1.04 (0.96-1.14)	1.01 (0.92-1.10)
	Scenario 3	u	1.04 (0.97-1.13)	0.99 (0.91-1.06)
		w	1.04 (0.96-1.12)	0.99 (0.91-1.07)
	Scenario 4	u	1.03 (0.96-1.10)	0.96 (0.89-1.04)
		w	1.03 (0.96-1.10)	0.97 (0.89-1.04)
**Net-equivalent household income (in CHF 1000)**[a]	Scenario 1	u	1.00 (0.87-1.15)	0.92 (0.80-1.07)
		w	1.00 (0.87-1.15)	0.90 (0.77-1.05)
	Scenario 2	u	1.00 (0.85-1.16)	0.91 (0.78-1.08)
		w	1.00 (0.85-1.18)	0.89 (0.75-1.06)
	Scenario 3	u	1.02 (0.88-1.17)	0.94 (0.81-1.08)
		w	1.02 (0.88-1.18)	0.92 (0.79-1.07)
	Scenario 4	u	0.94 (0.83-1.07)	0.90 (0.79-1.04)
		w	0.94 (0.83-1.06)	0.88 (0.76-1.02)
**Financial strain**[a]				
No financial strain	Reference		1.00	1.00
Slight strain	Scenario 1	u	1.44 (0.79-2.61)	1.91 (1.10-3.34)*
Massive strain		u	2.83 (1.26-6.38)*	**2.04 (0.90-4.59)**
Slight strain		w	1.50 (0.80-2.80)	2.13 (1.18-3.85)*
Massive strain		w	3.11 (1.34-7.23)**	2.47 (1.13-5.41)*
Slight strain	Scenario 2	u	1.73 (0.91-3.27)	2.28 (1.22-4.27)*
Massive strain		u	2.72 (0.93-3.40)*	**2.07 (0.81-5.27)**
Slight strain		w	1.77 (0.90-3.47)	2.43 (1.27-4.66)**
Massive strain		w	2.89 (1.09-7.66)*	**2.23 (0.94-5.30)**
Slight strain	Scenario 3	u	1.39 (0.78-2.49)	1.89 (1.09-3.28)*
Massive strain		u	2.71 (1.24-5.91)*	**1.92 (0.87-4.26)**
Slight strain		w	1.45 (0.79-2.67)	2.07 (1.17-3.68)*
Massive strain		w	3.07 (1.37-6.88)**	2.34 (1.09-5.03)*
Slight strain	Scenario 4	u	1.64 (0.98-2.74)	1.87 (1.10-3.17)*
Massive strain		u	2.73 (1.31-5.70)**	2.48 (1.15-5.36)*
Slight strain		w	1.68 (0.98-2.87)	2.17 (1.24-3.79)*
Massive strain		w	2.78 (1.32-5.88)**	2.98 (1.38-6.41)**
**Partner status**[b]				
Having a partner	Reference		1.00	1.00
Not having a partner	Scenario 1	u	1.56 (0.95-2.57)	1.72 (1.04-2.84)*
		w	1.62 (0.96-2.72)	2.04 (1.22-3.42)**
	Scenario 2	u	1.61 (0.95-2.73)	**1.68 (0.98-2.89)**
		w	1.58 (0.92-2.73)	2.03 (1.16-3.57)*
	Scenario 3	u	1.45 (0.89-2.37)	1.67 (1.02-2.75)*
		w	1.52 (0.91-2.53)	1.97 (1.18-3.29)**
	Scenario 4	u	1.47 (0.95-2.27)	1.63 (1.01-2.64)*
		w	1.48 (0.94-2.32)	1.92 (1.17-3.16)**
**Social contact frequency**[b]				
	Scenario 1	u	0.89 (0.83-0.96)**	0.94 (0.88-1.01)
		w	0.87 (0.81-0.94)***	0.95 (0.88-1.03)
	Scenario 2	u	0.90 (0.83-0.97)**	0.95 (0.87-1.02)
		w	0.87 (0.81-0.95)***	0.96 (0.88-1.04)
	Scenario 3	u	0.90 (0.84-0.97)**	0.94 (0.88-1.01)
		w	0.88 (0.82-0.95)***	0.96 (0.88-1.04)
	Scenario 4	u	0.92 (0.86-0.97)**	0.94 (0.88-1.01)
		w	0.91 (0.85-0.97)**	0.94 (0.88-1.02)
**Number of supportive relationships**[b]				
	Scenario 1	u	**0.85 (0.72-1.00)**	0.75 (0.64-0.89)**
		w	0.83 (0.70-0.98)*	0.75 (0.63-0.89)**
	Scenario 2	u	**0.85 (0.71-1.01)**	0.73 (0.60-0.89)**
		w	0.82 (0.70-0.97)*	0.73 (0.61-0.88)**
	Scenario 3	u	0.85 (0.73-1.00)*	0.76 (0.65-0.90)**
		w	0.83 (0.71-0.97)*	0.76 (0.64-0.90)**
	Scenario 4	u	**0.90 (0.78-1.03)**	0.76 (0.64-0.89)**
		w	**0.90 (0.78-1.03)**	0.75 (0.63-0.89)**
**Satisfaction with overall social support**[b]				
Satisfied	Reference		1.00	1.00
Less than satisfied	Scenario 1	u	**2.55 (1.08-6.03)***	**2.35 (1.08-6.03)***
		w	2.46 (0.95-6.37)	2.25 (0.95-5.32)
	Scenario 2	u	2.18 (0.92-5.17)	**3.11 (1.30-7.40)***
		w	2.12 (0.82-5.45)	**2.62 (1.08-6.37)***
	Scenario 3	u	**2.67 (1.18-6.09)***	**2.54 (1.13-5.75)***
		w	**2.62 (1.06-6.44)***	2.28 (0.97-5.39)
	Scenario 4	u	1.83 (0.82-4.08)	2.17 (0.97-4.83)
		w	1.75 (0.72-4.25)	1.97 (0.84-4.63)
**Satisfaction with family relationships**[b]				
Satisfied	Reference		1.00	1.00
Less than satisfied	Scenario 1	u	2.92 (1.62-5.27)***	2.44 (1.37-4.34)**
		w	3.21 (1.72-6.00)***	2.82 (1.56-5.07)***
	Scenario 2	u	3.02 (1.60-5.69)***	2.73 (1.44-5.18)**
		w	3.43 (1.76-6.70)***	2.99 (1.57-5.72)***
	Scenario 3	u	2.78 (1.57-4.93)***	2.37 (1.33-4.20)**
		w	2.94 (1.59-5.43)***	2.49 (1.38-4.51)**
	Scenario 4	u	2.34 (1.36-4.04)**	2.31 (1.32-4.04)**
		w	2.68 (1.51-4.74)***	2.52 (1.42-4.45)**

**Satisfaction with contacts to friends**[b]				
Satisfied	Reference		1.00	1.00
Less than satisfied	Scenario 1	u	4.68 (2.66-8.24)***	3.39 (1.93-5.95)***
		w	5.89 (3.27-10.62)***	3.58 (2.01-6.36)***
	Scenario 2	u	5.74 (3.12-10.54)***	4.12 (2.22-7.65)***
		w	7.58 (4.07-14.13)***	4.16 (2.23-7.78)***
	Scenario 3	u	4.55 (2.63-7.86)***	3.29 (1.88-5.75)***
		w	5.56 (3.15-9.84)***	3.34 (1.87-5.96)***
	Scenario 4	u	3.53 (2.06-6.06)***	3.24 (1.88-5.58)***
		w	4.27 (2.36-7.75)***	3.31 (1.88-5.84)***
**Satisfaction with partner relationship**[b, c]				
Satisfied	Reference		1.00	1.00
Less than satisfied	Scenario 1	u	5.71 (2.50-13.04)***	2.01 (0.91-4.47)
		w	5.53 (2.44-12.51)***	2.20 (0.98-4.96)
	Scenario 2	u	6.44 (2.60-15.93)***	**2.69 (1.11-6.52)***
		w	5.91 (2.36-14.78)***	**2.71 (1.09-6.71)***
	Scenario 3	u	5.28 (2.38-11.71)***	1.87 (0.85-4.14)
		w	4.86 (2.19-10.76)***	1.90 (0.85-4.26)
	Scenario 4	u	3.93 (1.90-8.15)***	2.08 (0.98-4.39)
		w	3.75 (1.77-7.92)***	2.15 (0.99-4.66)

* $p \leq 0.05$, ** $p \leq 0.01$, *** $p \leq 0.001$. *P* values from unrestricted fraction missing information tests for scenario 1, 3, and 4. *P* values from Wald χ^2 tests for scenario 2.
Scenario 1: Multiply imputed data for predictors and control variables, outcome variables full case; *Scenario 2*: Full cases for all included variables; *Scenario 3*: Multiply imputed data for predictors and control variables, replacement of missing values in outcomes by 'best case' values; *Scenario 4*: Multiply imputed data for predictors and control variables, replacement of missing values in outcomes by 'worst case' values.

[a] Adjusted for partner status, social contact frequency, number of supportive relationships, satisfaction with overall social support, with family relationships, with contacts to friends, and control variables (sex, age, level and degree of lesion, etiology, time since injury, chronic pain, paid employment). Not mutually adjusted for other socioeconomic variables.

[b] Adjusted for education, income, financial strain, and control variables. Not mutually adjusted for other social relationship variables.

[c] Subgroup analyses: Including only participants having a partner.

Note: Weighted analyses conducted with inverse probability weights. **Bold** results indicate deviations from the weighted scenario 1 (main results displayed in Table 3).
Abbreviations: *HADS-D*: Hospital Anxiety and Depression Scale, depression subscale; *MHI-5*: 5-item Mental Health Inventory of SF-36.

Table C. Sensitivity analyses of associations of socioeconomic conditions and social relationships with mental health, coefficients and 95% confidence intervals (95% CI) of tobit regressions.

		**General mental health** (MHI-5 score 0-100, higher values = better mental health)	**Depressive symptomatology** (HADS-D score 0-21, higher values = more depressive symptoms)
Number of observations		456	498
Effect sizes		*Coefficient (95% CI)*	*Coefficient (95% CI)*
**Socioeconomic conditions**			
**Years of formal education** (per year)	Model 1	0.14 (-0.51-0.80)	-0.06 (-0.19-0.06)
	Model 2	0.01 (-0.61-0.63)	-0.00 (-0.13-0.12)
	Model 3a	-0.03 (-0.53-0.48)	0.00 (-0.11-0.11)
**Net-equivalent household income** (per CHF 1000)	Model 1	0.62 (-0.33-1.58)	**-0.18 (-0.41-0.05)**
	Model 2	0.31 (-0.65-1.27)	-0.11 (-0.33-0.11)
	Model 3a	0.21 (-0.69-1.11)	-0.10 (-0.31-0.11)
**Financial strain**			
No financial strain	Reference	0.00	0.00
Slight strain	Model 1	-7.90 (-12.20- -3.61)***	1.73 (0.76-2.69)***
Massive strain		-19.34 (-25.97- -12.72)***	3.38 (1.98-4.78)***
Slight strain	Model 2	**-7.04 (-11.17- -2.91)****	1.55 (0.60-2.49)**
Massive strain		-17.67 (-24.24- -11.09)***	3.18 (1.87-4.49)***
Slight strain	Model 3a	**-5.63 (-9.43- -1.83)****	1.20 (0.31-2.09)**
Massive strain		-11.67 (-17.75- -5.59)***	1.88 (0.69-3.06)**
**Structural aspects of social relationships**			
**Partner status**			
Having a partner	Reference	0.00	0.00
Not having a partner	Model 1	-6.35 (-10.22--2.48)**	1.33 (0.44-2.21)**
	Model 2	-6.49 (-10.26--2.71)**	1.60 (0.77-2.44)***
	Model 3b	**-5.68 (-9.25--2.12)****	1.47 (0.66-2.28)***
**Social contacts frequency**	Model 1	0.88 (0.37-1.39)**	**-0.19 (-0.31--0.07)****
	Model 2	0.93 (0.44-1.42)***	**-0.14 (-0.26--0.02)***
	Model 3b	0.90 (0.44-1.35)***	**-0.13 (-0.25--0.01)***
**Number of supportive relationships**	Model 1	1.87 (0.83-2.91)**	-0.55 (-0.76--0.34)***
	Model 2	1.92 (0.87-2.98)**	-0.47 (-0.67--0.27)***
	Model 3b	1.78 (0.72-2.84)**	-0.44 (-0.64--0.23)***
**Functional aspects of social relationships**			
**Satisfaction with overall social support**			
Satisfied	Reference	0.00	0.00
Less than satisfied	Model 1	-12.27 (-20.61--3.92)*	2.54 (0.84-4.24)*
	Model 2	-11.31 (-19.26--3.36)*	2.20 (0.63-3.77)*
	Model 3b	**-8.49 (-16.22--0.75)***	**1.64 (0.09-3.20)***
**Satisfaction with family relationships**			
Satisfied	Reference	0.00	0.00
Less than satisfied	Model 1	-15.52 (-20.52--10.53)***	3.13 (2.04-4.22)***
	Model 2	-14.28 (-19.10--9.45)***	2.87 (1.83-3.91)***
	Model 3b	-11.28 (-15.90--6.66)***	2.35 (1.35-3.36)***
**Satisfaction with contacts to friends**			
Satisfied	Reference	0.00	0.00
Less than satisfied	Model 1	-18.09 (-22.58- -13.61)***	3.89 (2.81-4.97)***
	Model 2	-16.07 (-20.63- -11.52)***	3.44 (2.40- 4.49)***
	Model 3b	-14.01 (-18.50- -9.52)***	3.11 (2.07-4.15)***
**Satisfaction with partner relationship**			
Subgroup: Number of observations			
Satisfied	Reference	0.00	0.00
Less than satisfied	Model 1	-11.03 (-16.76- -5.31)***	10.13 (3.22-31.86)***
	Model 2	-12.21 (-17.48- -6.94)***	11.65 (3.64-37.32)***
	Model 3b	-9.93 (-15.60- -4.25)**	**7.37 (2.22-24.44)****

* $p \leq 0.05$; ** $p \leq 0.01$; *** $p \leq 0.001$. *P* values from unrestricted fraction missing information tests.
Model 1: Unadjusted.
Model 2: Adjusted for sex, age, level and degree of lesion, etiology, time since injury, chronic pain, paid employment. Not mutually adjusted for other socioeconomic or social relationship variables.
Model 3a: Model 2 additionally adjusted for functional and structural aspects of social relationships.
Model 3b: Model 2 additionally adjusted for socioeconomic conditions.
Note: Predictors imputed by multiple imputation, outcome variables full case only. All analyses weighted by inverse probability weights. **Bold** results indicate deviations from the main results displayed in Table 3.
Abbreviations: *HADS-D*: Hospital Anxiety and Depression Scale, depression subscale; *MHI-5*: 5-item Mental Health Inventory of SF-36.
